# 213 Maintaining physical activity through the use of digital tools for people with a long-term condition/s: A systematic review

**DOI:** 10.1093/eurpub/ckae114.086

**Published:** 2024-09-26

**Authors:** Suzanne Mc Donough, Aoife Stephenson, Chloe Grimmett, Ameera Khan, Emily Lait, Leah Rose McDonough, Georgia Tanner, Rob Argent, Sarah Howes

**Affiliations:** RCSI University of Medicine and Health Sciences, Ireland; School of Health Sciences, University of Southampton, Southampton, UK; RCSI University of Medicine and Health Sciences, Ireland; School of Health Sciences, University of Southampton, Southampton, UK; RCSI University of Medicine and Health Sciences, Ireland; Emergency Department, Mater Hospital, Dublin, Ireland; RCSI University of Medicine and Health Sciences, Ireland; RCSI University of Medicine and Health Sciences, Ireland; RCSI University of Medicine and Health Sciences, Ireland; Ulster University, Belfast, UK

## Abstract

**Purpose:**

People with long-term conditions (LTCs) are less active than the general population. The clinical and economic benefits of increasing physical activity (PA) in this population depend strongly on how long the effects can be maintained.^1^ It is vital we find effective, sustainable and scalable solutions to support people with LTCs to maintain PA long-term. This review aims to investigate the effectiveness of digital interventions for PA maintenance among adults with one or more LTC.

**Methods:**

A protocol was registered on Prospero (CRD42022299967). Searches were conducted in seven electronic databases, for randomised controlled trials (RTCs) including adults with ≥1 long-term conditions. Screening, data extraction and quality assessment was undertaken by two independent reviewers. Study methods, participant characteristics, intervention characteristics (digital/non-digital elements) and PA outcomes were extracted at intervention end and maintenance time points; data pooling and meta-analyses were planned.

**Results:**

Twenty three RCTs were included. The majority of digital interventions were web-based; around half included a sensing device (wearable/phone). All interventions, except two, included non-digital elements (supervised exercise classes, education/advice/health care practitioner support). Interventions frequently advised independent PA through a digital system or in daily life. None included all design features considered key in PA maintenance such as theory, habit formation, use of sensors for goal-setting/self-monitoring/feedback, and health coaching. Meta-analysis did not identify a significant effect on PA in favour of digital interventions for device-based outcomes (14 studies, n = 2210 participants, SMD=0.11, 95% CI -0.01, 0.24, I^2^=24.35, p = 0.08] and showed only a small effect for subjective outcomes (9 studies, 1592 participants, SMD=0.13, 95% CI 0.02, 0.23, I^2^=8.43, p = 0.02).

**Conclusions:**

We did not show that digital tools supported the maintenance of PA. However, there was a great deal of heterogeneity across trials with respect to digital/non-digital approaches/control comparisons making it challenging to pool our data. Our confidence in these findings is very low as only two RCTs were at low risk of bias. We therefore expect that findings may change as more robustly designed studies are published. We recommend that future digital interventions consider all key digital and non digital design features to support PA maintenance.

**Funding:**

HRCI-HRB-2022-010 and ARC Wessex ARC005.

